# Some Practical Points in the Diagnosis and Treatment of Acute and Chronic Aural Suppuration and Their Sequellae

**Published:** 1913-04

**Authors:** James F. McKernon

**Affiliations:** New York City


### Some Practical Points in the Diagnosis and Treatment of Acute and Chronic Aural Suppuration and Their Sequellae

By JAMES F. McKERNON, M. D.
New York City

(Concluded.)

As complicating acute and aural suppuration, we frequently encounter a thrombus of the sigmoid sinus, jugular bulb or internal jugular vein associated with one or all of these conditions the petrosal sinuses are not infrequently found involved.

These involvements of the blood current for clinical purposes can be spoken of under three headings:

First: Those that are typical. Second: Atypical. Third: In those cases where the jugular bulb and sinus adjacent are primarily involved without microscopic disease of the mastoid process.

In the Typical case, the symptom of most importance is that of temperature, and depends upon the amount of septic material entering the general circulation. If large in quantity, there is a sudden rise from normal to 104° F., 105° F., or 106° F. or higher, followed by a quick remission to near the normal point or even below. There may be one rise or several during a period of twenty-four hours, depending upon the rapidity with which the septic material is entering the general circulation.

If the patient is kept under observation for several days these fluctuations of temperature become more frequent, with greater variations. Chills, when present, are valuable aids in diagnosis. At times chilly sensations are present, and take the place of more definite chill. The pulse rate usually corresponds with the temperature. When high, it is rapid, when low, a little above the normal rate. Pain is not a frequent symptom, unless the phlebitis is associated with an extension into the jugular vein, or there is an infected chain of lymphatic glands adjacent to it.

Nausea and vomiting are not frequent until the later stages of the disease. Eye symptoms are present in only about one-quarter to one-third of the cases. Cerebration during the earlier stages of the disease is invariably normal, and formerly we were misled by this condition, as we expected the patient to exhibit cerebral manifestations. These are absent, however, except during the advanced stage of the disease. As the infection progresses there is loss of appetite, coated tongue and foul breath, with dry skin, which later becomes yellow, indicative of septic absorption.

The leucocyte and differential blood count are of value only as showing the amount of resistance of the patient, and the absorption taking place. Of more value from a diagnostic standpoint, is that of blood cultures, showing whether a bacteriemia is present or absent. Its presence is fairly positive proof of an infected thrombus, excluding, of course, all other suppurative conditions of the body.

The Atypical cases are those that follow a mastoid operation and progress favorably for a few days after the bone has been opened. A brief history of such a case from the clinical standpoint illustrates well the course of the disease in this type of infection.

Take a case of mastoiditis that has been operated upon from five to twelve days, the temperature, during this interval, following the usual course of a typical post-operative mastoid case. On the fifth or sixth day when the patient is seen, we find him restless, appetite variable, tongue slightly coated, pulse between 90 and 100, where the previous day it was between 70 and 80, and the temperature which was normal, has risen to 99° F. or 100° F., or thereabout. The next day the temperature will be from one to two degrees higher, with *no* marked remissions. There has been a change in the general condition of the patient. The restlessness is more pronounced. There is a distinct irritability, and a disinclination to take food. The tongue has become dry around the edges, with a thicker coating than the previous day. The pulse has increased in rapidity from 110 to 120 per minute. There is a complaint of general headache, not localized, and a feeling of nausea, and sleeplessness is a prominent symptom.

When seen the following day, the temperature has gradually risen to 104° F. or higher. The pulse is now ranging from 120 to 140 per minute, and shows a diminution in tension. The headache is more pronounced and the restlessness, sleeplessness and irritability are prominent features. The white, or gray coating on the tongue has now changed. It is brown and dry, like leather, and has a glazed centre.

If the disease progresses, we find within the next twenty-four hours the temperature has risen to 105° F., or higher. The pulse has become more rapid—140 to 150 per minute, showing a diminished volume and weaker, and with this condition an absolute refusal to take food. At this stage they become drowsy, and if aroused are exceedingly irritable and wish to be left undisturbed.

As the disease progresses, the temperature will practically become stationary, with no marked remissions. The pulse gradually becomes weaker and we have a patient presenting all the evidences of a general pyemia. A most valuable physical sign in these cases, from a diagnostic standpoint, is that of the bone overlying the sinus. For the first few days following the operation we find, on inspection of the mastoid cavity, every portion of it apparently doing well, and covered with granulations, except at one point, and this point is a portion of the bone overlying the sinus. Here the bone will be found darker in color than when it was first exposed at the time of the operation, and there will be no granulations found upon any part of it. In other words, we have a mastoid cavity showing all the evidence of repair, except this point of bone over a localized area of the sigmoid sinus.

If the infection progresses, we will find in three or four days the mastoid wound presenting a different picture. The parts that were formerly covered with healthy, firm, granulations have now become pale and flabby, with a tendency to soften and break down, while the point of bone over the sinus will remain dark in color and devoid of all reparative process.

If the sinus was exposed during the mastoid operation, we will find at this stage the dura covering it presenting a whitish or grayish appearance devoid of lustre, and at times covered with a plastic exudate. Blood examinations here are of equal value as when dealing with the typical cases.

When the jugular bulb and sinus are primarily involved they become so during the very early stages of an acute purulent otitis media, the pathway of infection being directly through the floor of the middle-ear to the bulb below, through one or more dehiscences, in this region that has persisted from birth.

The possibility of an infection occurring in this way has been demonstrated by the writer, who found that in 160 dissections in infants, ranging in age from birth to thirty days, that dehiscences were present in 28 7/-0 per cent., many of them being multiple. While it is not claimed that all of these openings remain permanent throughout life, we do believe that a certain proportion of them do retain their patency, as occasionally we find them on autopsy in adults.

The only diagnostic symptom in these cases is an unusually rapid rise in temperature from 99° F. or 100° F. to 104° F., 105° F., or even 106° F., or higher, and quite as sudden as the rise is a remission to normal or below. The temperature may remain low for several hours, and then quickly rise again to be followed by a rapid remission, and this may go on indefinitely until the end.

If the temperature range is allowed to repeat itself for several days, the clinical picture becomes one of general pyemia.

During the temperature remissions these patients feel remarkably well. This is a phase of the disease which the parents and those unaccustomed to viewing it, find hard to comprehend.

They see such an apparent improvement in the patients that they are misled as to the dangers existing, and often believe they are well on the road to recovery until the next temperature wave occurs.

While it is beyond the scope of such a paper as this to speak of the treatment in detail, I will mention here and there a point in the care of these patients.

First: Operate as soon as the diagnosis is made. Nothing is gained by delay and much may be lost. In the first and third class of cases, operate if possible during the remission of the temperature. By doing this there is less depression, and the system has a greater reactive power, and a more rapid convalescence will surely follow.

In operating upon the first and second class of cases, the rule usually followed is: If we find upon opening the sinus, a parietal or firm clot, obstructing the lumen of the sinus, showing no tendency to disintegration, we remove it, establishing a flow of blood from both the distal and proximal ends, and control the bleeding by packing gauze against the distal end, and into the proximal. If, however, we find upon opening the vessel, pus or a clot broken down or undergoing disintegration, we cease further operation, for the time being at this point, and proceed to expose, ligate and resect the internal jugular vein, together with any of its tributaries that are involved, or any diseased glands encountered during the dissection, and then proceed to evacuate the sinus contents above and establish our blood flow from the torcular end.

Frequently there will be quite a free hemorrhage from the bulb end, even after the vein below has been removed. Here the blood comes from our unobstructed petrosal sinuses.

In the primary bulb cases, especially in children, if no pus or broken down clot be encountered, operative measures usually cease, after the evacuation of the clot and the establishment of the blood current from the sinus end. Should they, in the days that follow, show evidence of a further pyemic condition, then the vein should be resected.

In otitis meningitis whether in cases suspected or those well developed, we have a valuable aid from a diagnostic standpoint, by a withdrawal and examination of the cerebro-spinal fluid. By its early withdrawal from the spinal canal, we are made acquainted with its quantity, as determined by the amount coming through the canal, showing whether it be in excess of the normal amount, and whether it shows evidence of increased pressure beyond the normal, as it is being drawn.

The character of the fluid as drawn may show us at once evidence of disease of the meninges. At times when drawn it appears microscopically normal, but again upon subjecting it to microscopic and bacteriologic examinations we frequently obtain early and valuable information as to the intra-cranial condition existing, and thereby are enabled to make an early diagnosis not only of meningitis but its type as well, when otherwise it would have been necessary to wait some time before the classical symptoms of the disease became sufficiently manifest for recognition.

Its examination is also frequently of value in a differnetial diagnosis between an typical brain abscess, encephalitis and meningitis. Lumbar puncture is also of a distinct value from the curative standpoint in ceses of serous meningitis, as its frequent performance diminishes the intra-cranial pressure, and relieves the venous stasis within the skull.

When it comes to the treatment of otitis meningitis there is, unfortunately, but little to say. Of the serous type, repeated lumbar punctures, the application of ice externally and the internal administration of drugs, such as the bromides, to lessen the existing nervous irritability, is about all that can be done.

In purulent meningitis of otitis origin, I know of no specific; cases have been reported as cured by subdural drainage, but these were in all probability localized purulent areas, lying closely adjacent to the introduction of the drains.

The introduction of urotropin into the spinal canal, after the withdrawal of all fluid under pressure, has met with some success in cases where the predominating infection was that of the streptococcus. A concentrated solution, consisting of about one-half the quantity of that withdrawn from the spinal canal is introduced through the canula, the dose ranging from 30 to 80 grains, depending on the quantity of cerebro-spinal fluid withdrawn. This dosage can be repeated once or twice during twenty-four hours, depending on the general condition of the patient. If the temperature be lower and the general symptoms show an abatement it is best to wait for twenty-four to thirty-six hours before another dose be given, and this should be smaller in quantity than the one preceding it.

The writer has treated six cases of purulent meningitis by this method with three recoveries, all of the streptococcus variety; two cases were treated when the infection was that of the straphyloccus. Here the treatment had no apparent effect in alleviating the disease. In the sixth case the infection was that of the diphlococcus intercelluaris meningitidis. Here no favorable result was observed in its administration.

Another case of the streptococci variety was seen in consultation with a colleague where the urotropin was given, one dose each day of fifty grains for four days with recovery. In these cases that recovered the cerebro-spinal fluid contained pus with large numbers of streptococci to the slide and pure cultures of the organism were grown from the fluid drawn from the spinal canal.

While, of course, we are unable to say that these cases cited were of the type of a general purulent meningitis, the presumptive evidence was that they were, inasmuch as they exhibited the true clinical picture of the disease, plus positive laboratory findings. They may, however, have been cases where only a localized area of the meninges was involved.

The operation as devised and practiced by Haynes of draining the meninges through the cisterna-magna for this disease seems rational, especially if done early, but up to the present time no recoveries have been reported. Possibly the reason that this operation has thus far been unsuccessful has been because all the cases so operated upon were well advanced in the disease prior to its performance.

For several years the otologist as well as others has been availing himself of laboratory aid in the diagnosis of certain forms of aural disease. Some of these are confirmatory only of the clinical evidence presented, while others are of real value in arriving at a positive diagnosis.

From a series of blood examinations in over 400 cases the writer believes that these examinations will show two things:

First: the resisting power of the patient as shown by the number of leucocytes present, which if over 15,000 shows a good resistance. If daily examinations show a steadily increasing number of white cells, we infer that the patient's resistance is improving, even though the inflammatory process is more pronounced.

If, on the other hand, there is a small number of leucocytes from the first, the patient's resistance to the disease is poor, and during the further progress of the disease, should they steadily decrease the resistance becomes still poorer, and frequently this is an indication for an urgent operation, or calls for one being done earlier than would otherwise be necessary.

The second point of value for the clinician is the polynuclear percentage which shows definitely the amount of absorption taking place in the system as a result of the existing suppurative process. The normal percentage in the adult varies from 55 to 70, in the young child from 42 to 60. In a suppurative lesion of the mastoid process without complications the polynuclear percentage ranges from 70 to 80, where adjacent structures are involved, as the blood current in the sinus or vein it ranges from 80 to 96 per cent. In other words, the higher the polynuclear percentage the greater the absorption, and the more urgent the demand for operative procedure, and this is especially true if frequent examinations show a steadily decreasing number of leucocytes.

If, however, repeated examinations show a decreasing polynuclear percentage, with a stationary or slowly decreasing leucocyte count, it is evident that the suppurative process is diminishing, and that nature is eliminating the poison from the system.

The relation between the leucocytosis and the polynuclear is the valuable feature. Increasing leucocytosis, with stationary or falling polynucloesis, indicates better resistance and less absorption. While decreasing leucocytosis, and increasing polymuclosis indicates less resistance and a greater absorption. We must bear in mind, however, that a suppurative process in a bone cavity does not exhibit as high a polynuclear percentage as when present in the soft tissues of the body. While this differential blood and leucocyte count are of value in determining the absorption and body resistance, they should by no means supplant the clinical picture as presented by the patient, but should be utilized as one of the links in the chain of diagnosis confirmatory of the clinical and physical signs exhibited.

Blood cultures have proven of valuable aid in determining the condition of the blood stream in phlebitis of the sinus, bulb and vein, and by availing ourselves of this aid we have frequently been enabled to make an earlier positive diagnosis of involvement of these structures, than if we had awaited a development of a majority of the symptoms which usually accompany this disease.

If the infection is of an active or virulent type of a rapidly growing organism, a well developed growth is usually at the end of eighteen hours, but if no growth can be demonstrated the culture should again be placed in the oven and subjected to a longer period of incubation, as a negative result does not at this time, necessarily mean that there is absence of a bacteremia. If at the end of forty-eight hours no growth can be demonstrated, it is probable that no bacteremia exists, though there are exceptions to the rule.

In two cases of the writer's a growth did not show in the active type of infection, and showed upon operation a firmly organized clot, with no evidence of disintegration.

Should the first blood culture be negative, and the clinical symptoms as exhibited by the patient continue, then other cultures should be taken, for a thrombus may be present in the sinus or bulb, and still not show in the blood by one or more cultures. The explanation of this is, that at the time the negative culture was taken there was no bacteria being thrown off from the phlebitic area on account of the firmly organized clot, but later culture in one instance until after fifty hours incubation, and in the second fifty-five hours had elapsed before the culture showed a positive result. Both of these cases were of the class when softening and disintegration took place, colonies of bacteria were loosened from the thrombotic area and thrown into the general circulation, and the blood extracted from the vein at this time will show a true bacteremia present, so that a negative culture with a continuance of pyemic symptoms of a low grade, does not mean that there is absence of involvement of the sinus, and repeated cultures should be made. While I do not wish to be understood as advocating waiting in all cases of suspected sinus disease for a positive blood culture before operating, I do, however, urge that a blood culture be taken and repeated if necessary, provided the clinical evidence as presented by the patient be not sufficient for a positive diagnosis. If the clinical picture be sufficient for such a diagnosis, then the culture is merely a confirmatory but most valuable aid.

My belief, based upon a large number of blood cultures, is that fewer operations on the sinus, either exploratory or complete, would be done were blood cultures made a part of the routine practice of the otologist in these suspected cases of sinus involvement.

We should remember, however, that a suppurative process existing in other parts of the body can and does give rise to a bacteremia of the blood current, and the existence of such a condition should be eliminated prior to localizing it in the sinus or bulb.

Want of space and the desire to no longer trespass upon your time compel me to bring this paper to a close. I have tried to speak in a general way of many phases of aural suppuration which must be of interest to the general practitioner as well as to the otologist, for the former sees many of these cases first, and it is to him in the future that will belong, to a marked degree, the credit of a decrease in the number of cases of chronic purulent ear disease, for if recognized and treated early an acute otitis need rarely reach the chronic stage.

When, however, this stage of middle-ear disease is encountered, no time should be lost in rectifying the pathological conditions existing, not alone to prevent the possibility of the occurrence of the complications under discussion, but what is of the utmost importance to the patient, to preserve his hearing.

62 West 52d Street, New York.

---

Two Police Women have been appointed in St. Paul.

